# FROM FRONT TO BACK SEAT DRIVER: INVESTIGATING DRIVING RETIREMENT DECISION-MAKING WITH THE 85+

**DOI:** 10.1093/geroni/igad104.3363

**Published:** 2023-12-21

**Authors:** Sophia Ashebir, Taylor Patskanick, Lisa D’Ambrosio, Joseph Coughlin

**Affiliations:** Massachusetts Institute of Technology, Cambridge, Massachusetts, United States; Massachusetts Institute of Technology, Cambridge, Massachusetts, United States; Massachusetts Institute of Technology, Cambridge, Massachusetts, United States; Massachusetts Institute of Technology, Cambridge, Massachusetts, United States

## Abstract

It is impossible to consider personal transportation in the United States without engaging with the topic of driving. Over 90% of U.S. households own cars, while 83% of people report they drive several times a week. A critical but often overlooked component of the driving narrative particularly among older adults is the decision of when or if to forfeit the car keys, a topic which is both complex and, for many individuals and families, a source of stress and conflict. With a rapidly growing population of the oldest of older adults, the 85+ age demographic, it is crucial to understand the attitudes and behaviors of this diverse age group regarding the experience of driving retirement. Utilizing four semi-structured focus groups (n=17) and a 48-item survey of original and adapted measures (n=34) with a panel of the 85+, this study sought to understand people’s perspectives on driving, how current drivers think about and plan for driving retirement – or not, and participants’ general perceptions and attitudes toward the future of technology and driving. The data suggest four different vignettes or transportation paths with respect to driving among this age group that intersect with themes of autonomy, the experience of physical aging, the role of family, and key inflection points. Implications of this research highlight the need for targeted solutions and materials for people at varying levels of acceptance with their driving stage and underscore the need for more research and greater involvement of different stakeholders in conversations around transportation and aging.

